# COVID‐19 and the *Mental Capacity Act* in care homes: Perspectives from capacity professionals

**DOI:** 10.1111/hsc.13747

**Published:** 2022-02-09

**Authors:** Margot Kuylen, Aaron Wyllie, Vivek Bhatt, Emily Fitton, Sabine Michalowski, Wayne Martin

**Affiliations:** ^1^ Essex Autonomy Project University of Essex Colchester UK; ^2^ School of Health and Social Care University of Essex Colchester UK; ^3^ School of Law University of Essex Colchester UK; ^4^ Faculty of Law, Economics and Governance Utrecht University Netherlands

**Keywords:** best interests, care homes, COVID‐19, human rights, mental capacity, *Mental Capacity Act* (England and Wales) 2005 (MCA), public health

## Abstract

This study explores the experiences of professionals who worked with care home residents with impaired mental capacity in England and Wales during the COVID‐19 pandemic. It explores (i) how competing risks were balanced and (ii) how the *Mental Capacity Act* (MCA) functioned in care homes under pandemic conditions, with particular focus on its associated Deprivations of Liberty Safeguards (DoLS) and Independent Mental Capacity Advocacy (IMCA) systems. Between March and May 2021, we held an online survey and five focus groups aimed at professionals who worked in or with care homes during the pandemic. The study explored issues pertaining to residents with impaired mental capacity, alongside several other topics on which we report elsewhere. For this paper, we filtered data to only include responses from ‘capacity professionals’. The resulting sample comprised 120 (out of 266) survey participants and 18 (out of 22) focus group participants. We performed manifest content analysis on the filtered data and found that (1) participants reported a ‘massive discrepancy’ between the ways different care homes balanced the risk of COVID‐19 infection with the risks associated with severe restrictions. (2) Some suggested this was due to vague guidance, as well as care home type and size. Participants told us the pandemic (3) obstructed smooth operation of statutory safeguards designed to protect residents’ human rights and (4) resulted in confusion about the remit of the MCA during a public health crisis. Our findings raise concerns about the impact of pandemic‐related measures upon care home residents with impaired mental capacity. We urge further exploration and analysis of (a) the variability and inconsistency of restrictions applied at care homes, (b) the strain placed on key safeguards associated with the MCA, (c) uncertainty about the remit of the MCA during a public health crisis and (d) the human rights implications hereof.

**What is known about this topic?**

- COVID‐19 has had a significant impact on care home residents
- including those with conditions associated with impaired mental capacity.
- There have been anecdotal media reports of a ‘postcode lottery’ of regional and local discrepancies in care home visiting restrictions during the pandemic.
- There have been concerns about the pandemic rendering ‘all but unusable’ legal frameworks like the MCA (Ruck Keene, 2021, p. 8).

**What this paper adds?**

- Evidence of the pandemic's impact on the operation of the MCA’s DoLS and IMCA systems.
- Evidence of confusion among capacity professionals regarding the legal standards to apply in determining whether restrictive measures comply with human rights standards.
- Empirical evidence of variations in how visiting and other restrictions were managed across different care homes and factors associated with these differences

## 1 INTRODUCTION

The impact of COVID‐19 on care homes across the United Kingdom has been well documented. By May 2021, there had been 27,179 excess deaths among care home residents since the onset of the pandemic (Dunn et al., 2021). The significant mortality associated with COVID‐19 in care homes led to implementation of restrictive measures aimed at limiting infection, including suspension of visits for families and friends, limits on residents’ ability to leave the facility, and isolation of residents within individual rooms or areas (Department of Health & Social Care, 2021). While aimed at protecting individual residents as well as the wider care community from COVID‐19, these measures also negatively impacted resident health and well‐being. For example, visitation restrictions in care homes have been linked to reduced nutritional intake and weight loss, loneliness and isolation and reduced quality of life (Hugelius et al., 2021; Pérez‐Rodríguez et al., 2021; Sizoo et al., 2020; Wammes et al., 2020). In addition to the crisis of high COVID‐19 mortality, care home staff have, therefore, faced what Chu et al. (2020) refer to as a ‘competing crisis’: the deleterious physical and psychological effects of social isolation associated with severe restrictions (Amnesty International, 2020; Vicary et al., 2020).

Restrictive measures present a distinctive set of challenges when they affect residents whose decision‐making abilities are impaired, who lack mental capacity to consent to restrictions, or for whom conditions such as dementia or learning disabilities may make compliance difficult. These challenges can themselves produce additional risks for such residents, for example increasing the risk of infection spreading among residents who are unable or unwilling to self‐isolate. This is significant considering that conditions associated with impaired mental capacity, such as dementias and learning disabilities, are prevalent in care settings (Gordon et al., 2014; Public Health England, 2016). This is complicated further by evidence of these conditions being linked to an increased vulnerability to adverse effects stemming from restrictions (Alzheimer’s Society, 2020; Brown et al., 2020; Courtenay & Perera, 2020; Theis et al., 2021; Velayudhan et al., 2020) and that these residents often have specific needs that may be difficult to meet while restrictive measures are in place. This in turn can compound risks of harm when restrictions are implemented. Little is known about the way care homes have managed the competing risks of protecting these vulnerable residents’ overall health and well‐being, while simultaneously protecting them and the wider care community against COVID‐19 (Liu et al., 2021).

Concerns have also been raised about the impact of the pandemic on the human rights of residents with impaired mental capacity (Wilson, 2020). In England and Wales, decisions made on behalf of people with impaired capacity are regulated by the *Mental Capacity Act* (MCA, Office of Public Section Information (OPSI), 2005), which is associated with two key instruments for protecting their human rights. The first is the ‘Deprivation of Liberty Safeguards’ (DoLS) (Ministry of Justice, 2008). The purpose of the DoLS system is to ensure that any deprivation of liberty in care home settings is in the person's best interests and is necessary and proportionate to the risk of harm. Best Interests Assessors (BIAs) and Relevant Person's Representatives (RPRs) have statutory responsibilities in implementing the DoLS system and, therefore, play a vital role in the protection of human rights in care settings.^1^ A second instrument is the advocacy system associated with the MCA. Independent Mental Capacity Advocates (IMCAs) play an important role in ensuring that, if someone with impaired mental capacity has no appropriate person to advocate on their behalf, they can still participate as fully as possible in decisions about serious medical treatment or accommodation and that such decisions are informed by their wishes and feelings, beliefs and values (MCA 2005, ss. 35‐41). Where a best‐interest decision needs to be made in relation to accommodation or serious medical treatment for a resident with impaired mental capacity and without an appropriate advocate, an IMCA must be instructed. If a care home resident is deprived of their liberty, then a DoLS assessment must be undertaken.

This paper reports on the experiences of professionals who have worked with care homes in England and Wales during COVID‐19, who have either been physically present within care homes or have worked with them remotely (e.g. conducting remote DoLS assessments). Specifically, it focuses on the experiences of those we will refer to as ‘capacity professionals’: that is, professionals such as BIAs, IMCAs and DoLS practitioners whose professional role requires them to have specialist understanding of mental capacity. This cohort includes care home managers. These professionals may or may not be involved in the day‐to‐day care of residents, but they have specific insights into the way restrictions have been handled when residents lacked the mental capacity to consent to them, and into the operation of the MCA (especially the DoLS and IMCA systems) during the pandemic. They also play an important role in protecting and promoting the human rights of residents with impaired mental capacity, for example by referring potential violations of those rights to relevant authorities, including Local Authorities and the courts.

The aim of our study was to gather information about the management of pandemic restrictions in care homes where at least some residents lacked mental capacity to make significant decisions relating to the pandemic response. How were restrictions handled when residents were unable to consent to them, and how did the pandemic affect the operation of the MCA and its associated safeguards? We set out to capture reports on these matters from capacity professionals who were themselves working either in or with care homes during the pandemic, and in this way to build up a picture of the relevant practices. To be clear, our aims in this paper are *neither comparative nor evaluative*. We do not set out to compare the situation of these residents to that of other residents or to the rest of the population. We do not attempt to evaluate the guidelines provided to care homes, and we do not attempt to determine whether the right balance was struck in the implementation of restrictive measures. These are all matters that merit further attention, but for present purposes our aim is more modest: we rely on well‐placed professionals in specific statutory roles to help build up a record of what transpired when the provisions of the MCA were applied under unprecedented circumstances.

## 2 METHODS

This article draws on survey and focus group data collected as part of a larger study investigating the impact of COVID‐19 on human rights in care homes in England and Wales. For the purposes of this article, we filtered data relevant to the focus of the article. This section provides some general information about the overall study and explains our approach to filtering and analysing the data.

### 2.1 Sampling

For the larger study, purposive sampling was used to survey health and care professionals who had worked in or with care homes since the onset of the COVID‐19 pandemic. We recruited across the breadth of professions involved in either the direct care of residents within care homes, or external professionals involved in supporting care home functions. We sought geographic representation across different areas of England and Wales. We recorded 262 survey responses in total, and recruited a total of 22 focus group participants.

For the purposes of this article, we filtered survey and focus group data to include only responses from capacity professionals. We defined capacity professionals as those with specialist knowledge of mental capacity. This filtering process involved reviewing participant roles for both the survey and focus groups and compiling a list of roles to be included in the study.^2^ The filtered data included responses by 120 survey participants and 18 focus group participants. An overview of the sub‐sample composition for the survey and focus groups can be found in Tables 1 and 2. In‐text quotations are coded by data source (survey or focus group) and professional role. Codes can be found in Tables 1 and 2.

**TABLE 1:** Survey subsample composition

Survey	
*Total*	*120*
Region	
England	114
South East	23
Yorkshire and the Humber	21
London	19
North West	15
South West	12
East of England	8
West Midlands	7
East Midlands	6
North East	3
Wales	5
Role*	
Best Interests Assessor (S‐BIA)	62
Social worker (S‐SOC)	32
Advocate (S‐ADV)	55
Other (S‐OTH)	10

* Participants could pick more than one role.

**TABLE 2:** Focus group subsample composition

Focus groups	
*Total*	*18*
Role	
Best Interests Assessor (FG‐BIA)	7
Advocate/Advocacy Manager (FG‐ADV)	4
DoLS Practitioner or Manager (FG‐DoLS)	2
MCA Lead (FG‐ML)	1
Residential Services Manager (FG‐RSM)	2
Support Worker (FG‐SUP)	2

### 2.2 Recruitment

A link to the online survey and a Survey Participant Information Sheet was sent via email to existing networks of the research team members and two allied health practice research networks with whom authors had a prior involvement and shared via social media. Informed consent was obtained via tick box on the Qualtrics online platform.

At the end of the survey, participants were asked to provide an email address if they were interested in participating in a focus group aimed at further exploring topics in the survey. Collected email addresses were not connected to any survey answers. An invitation to the focus groups with a link to a Participant Information Sheet was sent out to these email addresses; invitees were asked to return a signed consent form via email and indicate their availability if they wished to participate. Focus groups were formed based on availability.

### 2.3 Study design

We adopted a sequential explanatory mixed methods design (Ivankova et al., 2006) consisting of an online survey followed by focus groups aimed at providing context and depth to our survey findings. Following the pragmatic paradigm, we aimed to generate practical insights using methods best suited to our study aims, without commitment to a single underlying philosophy or ontological position (Morgan, 2014). The survey was constructed using Qualtrics software. Survey topics were informed by a scoping review and analysis of registration data and delegate feedback from a series of ‘rapid‐response to COVID‐19’ webinars held in collaboration with the National Mental Capacity Forum. Topics included the use of restrictive measures, the role of different forms of guidance (e.g. from government departments, local authorities and professional bodies), access to care for residents, Do Not Attempt Cardio‐Pulmonary Resuscitation (DNACPR) orders and the use of and the role of IMCAs during the pandemic.^3^ Survey questions were developed through discussion among members of the research team and seven external partners with expertise across law, mental capacity legislation and social care. A pilot survey was reviewed by these external partners. The final survey comprised mainly closed‐ended questions, with some opportunities for elaboration in free text response. It took 20–30 min to complete. The entire survey included 53 questions, but a branching structure was used which directed any individual respondent only to a subset of questions. For the survey instrument, see Appendix A.

A focus group protocol (Appendix B) was developed and refined through discussion with the research team, in line with key issues identified in the survey findings: access to care, use of restrictive measures, the use of DNACPR orders and the use of IMCAs. Five focus groups of approximately 1.5 hr were held online; each group had between three and five participants (excluding attending members of the research team). They were transcribed for analysis.

Ethical approval for this study was obtained from the University of Essex Humanities Sub‐Committee.

### 2.4 Data collection

Survey data were anonymous; no personally identifiable information was collected. Focus groups were confidential and commenced with an outline of privacy and confidentiality expectations, and a description of the data storage practices. To protect confidentiality, participants were asked not to identify their employer, other professionals or service users. Focus group transcripts were anonymised. Anonymous survey responses and anonymised focus group transcripts were stored on a secure drive, with access restricted to members of the research team.

### 2.5 Data analysis

Following the filtering process, quantitative survey data were analysed with simple descriptive statistics computed via the Qualtrics survey platform. Free text survey responses and focus group transcripts were extracted into the QSR NVivo platform and analysed using a manifest content analysis approach: we focused on the ‘visible and obvious’ meaning conveyed by participants, rather than seeking an in‐depth interpretation of underlying or hidden meaning (Bengtsson, 2016, p. 10). This approach aims to stay close to the original meaning conveyed by participants and is an appropriate strategy when seeking to understand experiences or perceptions of a defined phenomenon (Vaismoradi et al., 2013).

Our approach reflected the four‐stage process described by Bengtsson (2016, p. 10): two members of the research team read through the collated focus group transcripts and free‐text survey responses to develop familiarity with the data. Using a deductive approach, an initial ‘open coding’ process was undertaken using two initial categories that reflected content areas of the dataset most relevant to this article: the handling of restrictions and the operation of the MCA during the pandemic. Several processes of refinement through discussion resulted in five sub‐categories, with consensus among all authors that this structure provided a comprehensive coverage of content most relevant to our study aims. These five sub‐categories were used to structure the article during compilation and write‐up; in keeping with a manifest analysis, selected verbatim quotations from participants were drawn on significantly during this stage, and feature prominently in the presentation of findings (Bengtsson, 2016).

## 3 FINDINGS

### 3.1 Managing competing risks

**FIGURE 1:**
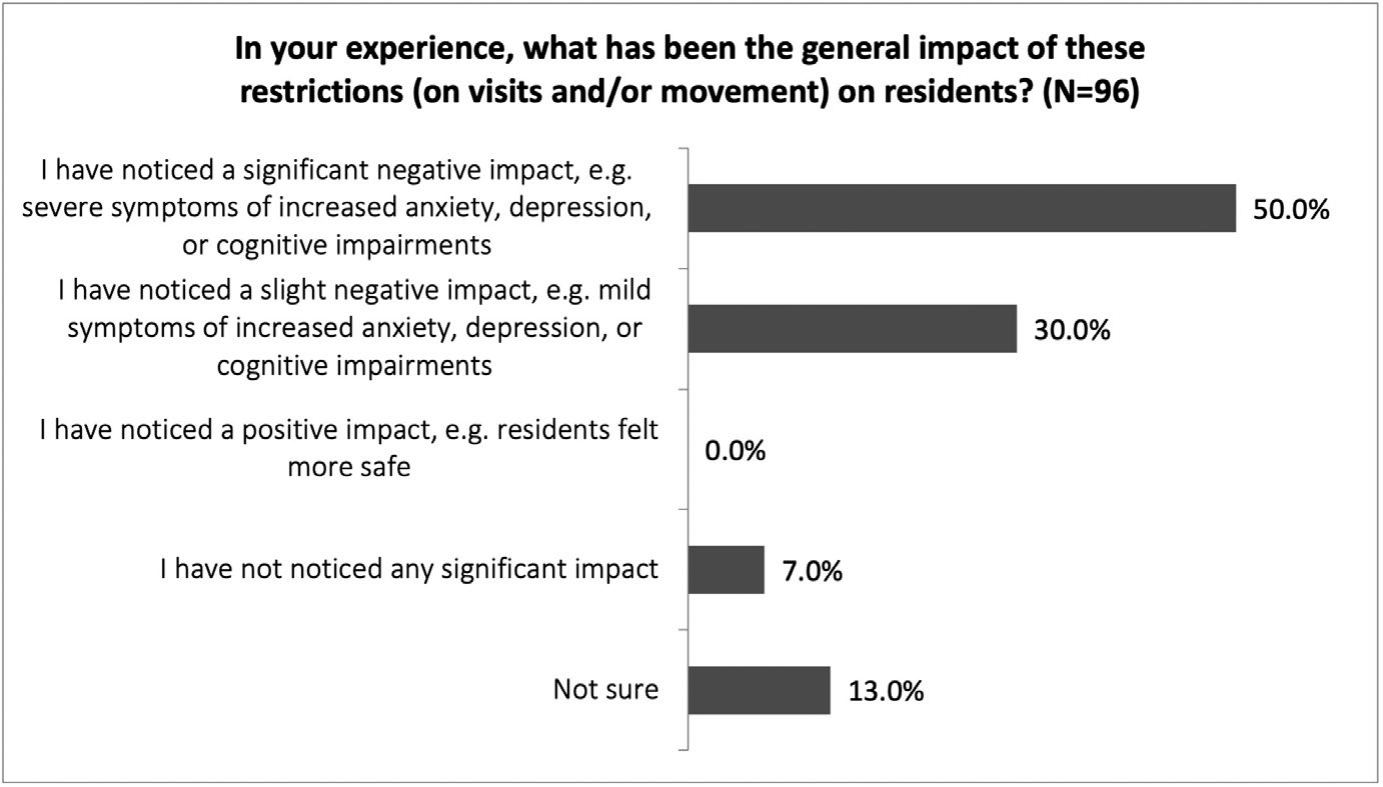
This figure shows filtered survey responses by capacity professionals to the question: ‘In your experience, what has been the general impact of these restrictions (on visits and/or movement) on residents’? Respondents could select one answer only. A majority of respondents noticed a negative impact, be it severe (50%) or slight (30%)

More than 80% of capacity professionals in our survey reported negative impacts on residents of restrictions on movement and visits to care homes. Reported impacts included increased anxiety, depression, or cognitive impairment (see Figure 1). Some participants noted that restrictions had ‘significantly impacted’ on care home residents with learning disabilities (S‐OTH1). Participants also reported increased self‐harming behaviours, boredom and reduced physical activity resulting in pressure ulcers and chest infections. Participants also gave examples of how restrictions were handled when residents had impaired mental capacity, reporting significant discrepancies between the ways different care homes managed restrictions and identifying three potential reasons for these discrepancies, discussed below.

#### 3.1.1 Discrepancy

Participants reported a ‘massive discrepancy’ (FG‐BIA1) in the ways different care homes balanced the risk of infection with the risks associated with severe restrictions. Some care homes were described as ‘creative’ (FG‐ADV1) in the way they implemented infection control measures, which was reflected in the way measures were implemented for residents with impaired mental capacity. For example, one participant told us about a care home resident with dementia who was required to isolate for 14 days:

This lady did not want to stay in her room and she left…The care home didn’t…help her or support her to return to her room coercively, she enjoys spending time in the conservatory…so what they did was…set it up just for her. So she freely went from her room to the conservatory, and they supported her to isolate in a communal area for herself. (FG‐DoLS1)

One senior support worker reported that a resident was allowed to wander despite having tested positive for COVID‐19, arguing that ‘it's not a secure unit, it's their home; you have to work with them as opposed to impose on them’ (FG‐SUP1).

In contrast, other care homes were perceived as more narrowly focused on protecting against infection. In this context, some professionals gave examples of care homes taking a blanket approach to restrictions, that is, applying them across the care home without undertaking individualised assessments. In some instances, this led to restrictions that professionals perceived as poorly tailored to resident needs:

I’m RPR for a lady…with really high sensory needs, and before lockdown she was… really active. All that stopped in lockdown. In the first few months, she put on three to four stone, her behaviours…became really challenging and I’d see in her records some…3‐to‐1 staff restraint, when she was attacking other service users, PRN lorazepam used…five six times a week, sometimes multiple times a day…I asked the care home manager if she could go for a walk in the park with staff…and I was told…that there's an absolute blanket ban on anybody leaving the grounds, apart from medical reasons. (FG‐ADV1)

Overall, professionals reported on divergent approaches to how restrictions were managed and implemented for residents at risk of impaired mental capacity:

There’s somebody with a learning disability, they just wouldn't even do a dynamic risk assessment, no matter how many times we asked them, [even though] that was in the guidance…He’s registered blind and they suggested window visits…It was such a contrast to another home, where there was a lady with dementia who was blind, and they made an exception… her husband could come in and hold her hand in a safe environment. …one home would do things…in a personalized way, but other homes would just have blanket bans on anybody doing anything. (FG‐ADV2)

#### 3.1.2 Reasons for discrepancy

Participants suggested several factors that may have contributed to this discrepancy‐handling restrictions. Some participants told us guidance ‘has not been specific enough’ (S‐BIA1), inviting different interpretations and resulting in divergent approaches:

[I] have assessed residents in care homes that [had] wide‐ranging response[s]. The concern I had was for residents that had harsher restrictions the vague guidance could still be used to cover this… (S‐BIA1)

The guidance the Government gave were very vague…I remember doing two assessments with two homes, both doing two completely different things – one kind of more focusing on human rights of the residents… the other one…very restrictive. But…when I then checked on the guidance…the guidance kind of fit each home, because the government guidance was so vague…and care homes kind of interpreted it how they wanted. (FG‐BIA3)

Some professionals also perceived learning disability services to be more inclined towards a ‘flexible’ approach to imposing restrictions, and ‘more proactive in terms of finding solutions’ (FG‐BIA3) as compared with older people's care homes:

I have found that residential homes and nursing homes for the elderly seem to be much more restrictive, whereas…the homes for people with learning disabilities have been little bit more lenient and a bit more realistic about the restrictions, more flexible and allowing the residents to be moving within the communal areas rather than confined to their rooms… (FG‐BIA4)

Care home size was a third factor perceived to shape how restrictions were imposed, with some participants describing smaller care homes as more likely to take an individualised approach to managing risks and imposing restrictions:

I think the smaller run care homes seem to be a bit more flexible…The larger organizational care homes, I find to be more blanket and more restrictive, because they were very much going by ‘this is what our head officer said’. Whereas the smaller care homes were kind of going, ‘this is how we find a way, this is how we've interpreted this…’ (FG‐BIA3)

### 3.2 Navigating the Mental Capacity Act during the pandemic

We asked participants about the impact of the pandemic on the operation of the MCA and the associated DoLS and IMCA systems. Our findings indicate that (1) visiting restrictions significantly complicated the workings of these systems and that (2) there was uncertainty as to whether and how the MCA applied during a public health crisis. Some participants told us that (3) there may be longer‐standing issues with knowledge of the MCA among some care professionals.

#### 3.2.1 The Mental Capacity Act during the pandemic

Although participants described their continued efforts to execute their responsibilities in difficult and changing circumstances, it was clear that the pandemic significantly complicated the functioning of the DoLS and the IMCA system.

##### The DoLS system

**FIGURE 2:**
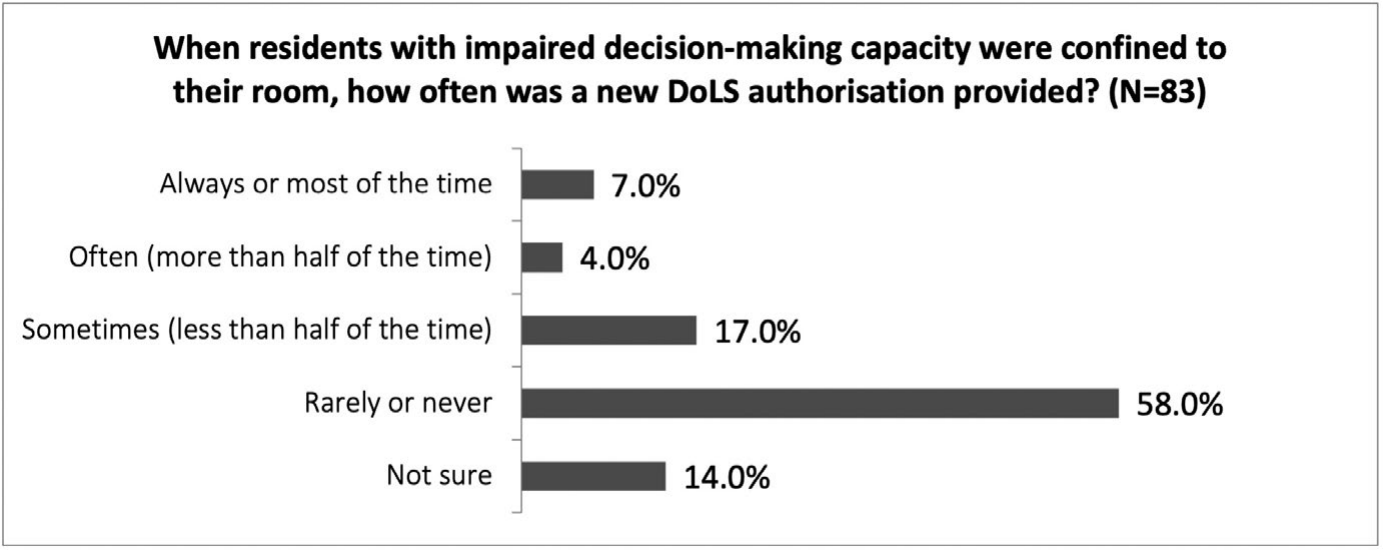
This figure shows filtered survey responses by capacity professionals to the question: ‘When residents with impaired mental capacity were confined to their room, how often was a new DoLS authorisation provided’? Respondents could select one answer only. A majority of respondents (58%) said new authorisations were rarely or never provided

Both our survey and focus groups showed that a new DoLS authorisation was rarely requested when residents with impaired mental capacity were restricted to their room (see Figure 2).

**FIGURE 3:**
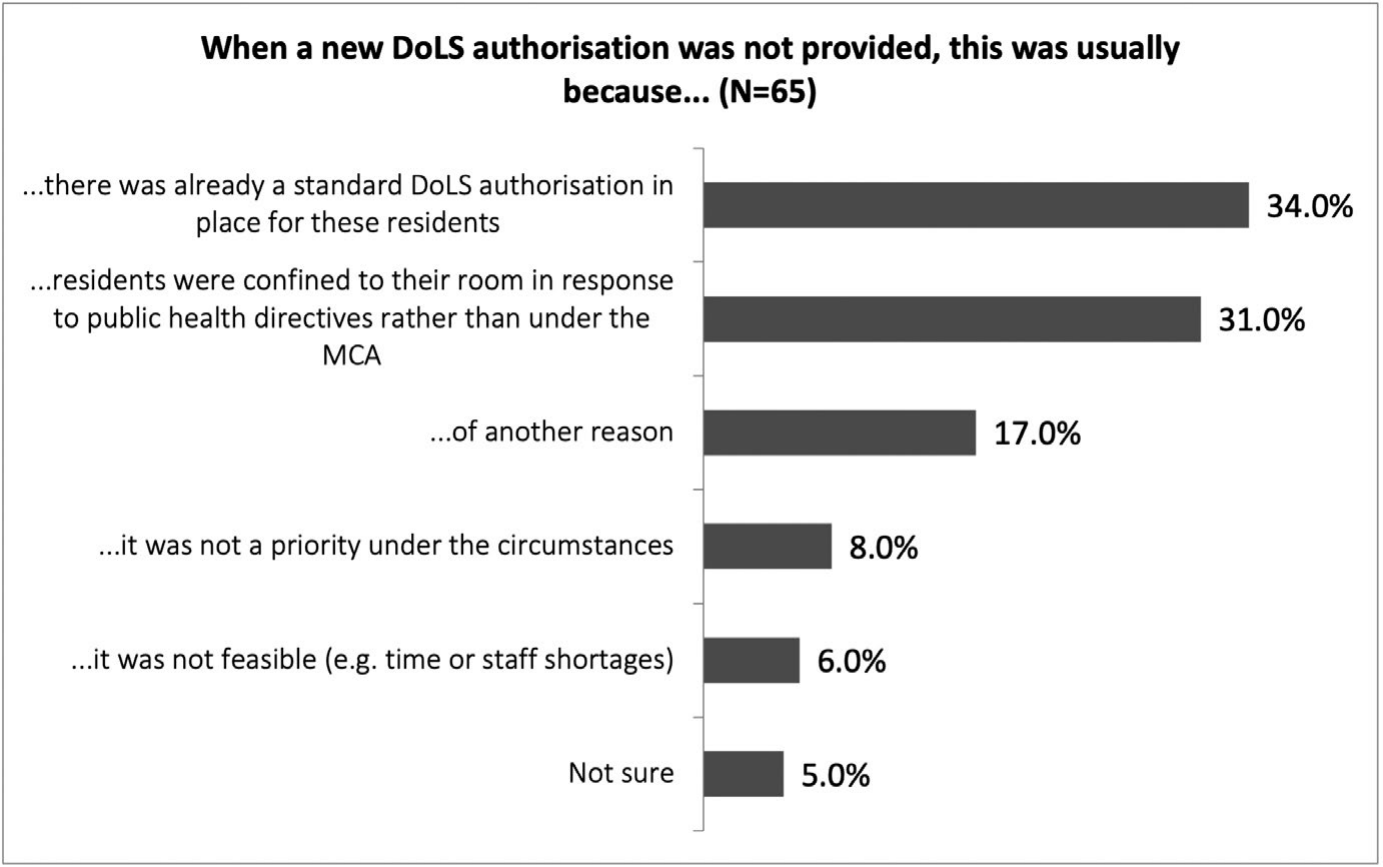
This figure shows filtered survey responses by capacity professionals to the question: ‘When a new DoLS authorisation was not provided, this was usually because...’ Respondents could select one answer only. A majority of respondents responded that there was already a standard DoLS authorisation in place for these residents (34%) or that residents were confined to their room in response to public health directives rather than under the MCA (31%)

In part, this was attributed to practical obstacles, with some survey participants saying that completing new authorisations was not feasible, or not a priority, under the circumstances (see Figure 3). These considerations were further emphasised by focus group participants:

We’ve always found there’s been a quite a backlog anyway, with DoLS, it can take quite a very long time… We didn’t put fresh DoLS in for people…when they were having to isolate for 14 days. But imagine if we did…I think by the time somebody came out, they would have been out of isolation anyway. …[W]e wanted to use our time in the best way we could, and that was to make sure that the people who we’re supporting had everything that they needed, not filling in a load of paperwork to not hear anything back. (FG‐RSM1)

##### The independent Mental Capacity Advocacy system

Participants told us the MCA’s advocacy system had also operated less effectively during COVID‐19. For example, some professionals described difficulties contacting care homes or having to initiate contact in circumstances where care homes would ordinarily do so:

I am very concerned about the inability to access people during the pandemic. Some homes have not answered the phones, have refused to accept visits and even when homes have passed on information it is hard to do advocacy without visiting or accessing records for yourself. I am very worried that bad practice may have developed in homes without outside scrutiny. (S‐ADV1)

As an advocate I have not been approached by care homes for BID [Best Interests Decision] making. I have had to point out to care homes where BID are needed and request them… (S‐ADV2)

Furthermore, capacity professionals noted that remote working, which was commonplace during the pandemic, was a significant challenge:

Contacting care homes to speak with unit staff remotely in some of the care homes can be very frustrating compared to face to face visits…You are unable to view the evidence of the information given to you on the phone. (S‐ADV3)

The RPR role has been difficult in those clients who have cognitive or sensory impairments where communication is not just verbal. (S‐ADV5)

My experience is that advocates have supported best‐interests decisions, but COVID restrictions have made involvement of the person, and consultation with family more difficult and on occasion uncertain. (S‐ADV6)

Finally, some participants told us they struggled to access care homes even when they deemed a visit necessary:

I found that care homes have been very reluctant to accept visits even when I have deemed it as urgent and essential to the person I am supporting…(S‐ADV7)

Here, again, participants reported discrepancies. For example, one respondent said that some care homes were ‘perfectly happy to allow the visits and allow the assessments because they have done the risk assessments and created …methods to be able to do that safely’, whereas at other homes, there was ‘just no question: you're not coming in’ (FG‐BIA4).

#### 3.2.2 Remit of the Mental Capacity Act during a public health crisis

Participants reported on a lack of clarity about how the MCA interacted with public health guidelines. They told us some care homes did not consider the MCA to be a relevant framework when implementing restrictions in the interest of public health:

In my experience care homes have not seen anything relating to the pandemic as a best‐interests decision but as an instruction from the gov[ernmen]t…. (S‐ADV2)

As an advocate I have seen so many decisions made due to ‘risk assessments’ or ‘covid guidance’ and no use of the MCA… (S‐ADV2)

Some participants noted a recurring, if false, assumption that easements to the MCA had been introduced:

I think three different members of staff of that home said, ‘well, they've done easements to the Care Act, they’ve probably done them to the Mental Capacity Acts as well.’ (FG‐ADV2)

I have had a number of care homes telling me that there were easements to the MCA, when there never were. (S‐ADV2)

This lack of clarity was also reflected in the fact that new DoLS authorisations were rarely requested when residents were deprived of their liberty to prevent COVID‐19 infection. 31% of survey respondents said new authorisations were *not* provided because residents were confined to their room in response to public health directives rather than under the MCA (see Figure 3). During the focus groups, several participants told us care homes saw the COVID‐19 restrictions as an altogether different issue than deprivations of liberty under the MCA, assuming ‘it was a public health thing because it was about risk to others’ (FG‐BIA5).

One DoLS Practitioner told us it was not always possible to remain MCA‐compliant when imposing public health restrictions but that they tried to operate within the ‘spirit’ of the MCA:

A key part of my role is: ‘…what's the legal framework for this, what are we relying on here for authority to do whatever it is we think we should do?’, and I’ve been conscious of fudging that to the limits and beyond…we know we've got to restrict people's movements, because otherwise they'll get COVID or they'll give COVID to other people, but trying to pick your way through the Mental Capacity Act and public health regulations and find…the justification for it…There's a point beyond which you’re just…you're on your own…What I’ve done…is to say, well, so long as we're doing it in the right spirit, …that's the best that we can do, but we are actually going beyond what you can actually really do under the Mental Capacity [Act] and all the other…legal frameworks. (FG‐DoLS2)

This uncertainty as to whether COVID‐19 restrictions fall within the remit of the MCA and DoLS, one participant noted, made it difficult for capacity professionals to challenge any decisions they felt were a potential breach of residents’ rights under the MCA.

#### 3.2.3 Knowledge of the Mental Capacity Act

A final finding is that respondents linked inconsistent adherence to the MCA during the pandemic with a broader, and longer‐standing, lack of understanding of the MCA among certain care professionals. As one survey respondent noted, ‘knowledge from staff (including managers) of the *MCA* and DoLS is often low (S‐ADV8)’:

the major concern that has been raised for us is that care homes have not understood capacity well—how to carry out an assessment properly, have not been referring for IMCAs, that capacity is decision‐specific, and have not understood that family cannot give consent for medical decisions without an LPA for health and welfare in place, or that a restriction to a room is not covered by a previous DoLS authorisation. (S‐ADV9)

In contrast, one participant stated that ‘social workers generally have a really good understanding of the MCA and the role of an IMCA’ (FG‐ADV1). This suggests that varied knowledge of the MCA in different professional groups may have been a barrier to effective and consistent application of the MCA, both before and during the pandemic.

## 4 DISCUSSION

Our findings provide evidence of significant differences in how different care homes implemented restrictions when residents were unable to consent to them, which may in part be due to factors like care home size and type. The pandemic also obstructed smooth operation of the MCA and created confusion as to the remit of the MCA during a public health crisis. Finally, variable levels of knowledge of the MCA among care professionals may have shaped the management of restrictions.

Our findings are broadly consistent with a developing body of literature documenting the impact of restrictive measures on residents with conditions associated with impaired mental capacity (Alzheimer’s Society, 2020; Brown et al., 2020; O'Caoimh et al., 2020). Our findings also reveal that capacity professionals experienced significant variations in the way different care homes interpreted and enforced restrictive measures, complementing media reports of a ‘postcode lottery’ of regional and local discrepancies in restrictions to face‐to‐face visits (Tapper, 2020). Moreover, capacity professionals in our study suggested additional factors potentially contributing to differences in how guidance was interpreted and implemented. Smaller care homes, and those catering for adults with learning disabilities as opposed to older people, were perceived by some as being more flexible in their approach and more likely to assess and manage risks on an individual basis. Although our data preclude any firm conclusions about the correlation between restrictions imposed and care home size or type, this points to further avenues of research investigating relevant factors explaining divergent responses to COVID‐19 in care homes (Marshall et al., 2021).

Furthermore, our findings highlight challenges faced by capacity professionals during the pandemic. Pressures of managing the pandemic in care homes, and the impact of restrictions on routine access for capacity professionals, at times obstructed the DoLS and advocacy systems. Remote assessments sometimes made it difficult to verify relevant information or compromised effective communication for those with impaired mental capacity. These findings confirm concerns that the pandemic would inhibit optimal best‐interest decision‐making (Parsons & Johal, 2020) and raises concerns about proposals to continue virtual safeguarding practices beyond the pandemic (Anka et al., 2020).

Our findings document uncertainty among professionals as to the role of the MCA during a public health crisis. Early in the pandemic, Ruck Keene expressed his concern that the pandemic had ‘apparently rendered all but unusable’ legal frameworks like the MCA (Ruck Keene, 2020, p. 8). Although the MCA continued to be used in locked‐down care homes, our findings confirm that he was right to sound the alarm. Participants in our study reported that some care homes considered obligations under the MCA to be secondary to, or even superseded by, public health and infection control guidance. We also found renewed uncertainty among professionals as to when a restrictive measure engages the DoLS system.^4^

Our findings have human rights implications. Restrictive measures in care homes engage fundamental human rights, including rights concerning liberty, privacy, family life and non‐discrimination. This is not the place to provide a detailed human rights analysis, but we note two concerns that emerge from these findings. The first pertains to the DoLS and IMCA provisions associated with the MCA. These provisions form a crucial part of the domestic framework for protecting the human rights of care home residents, yet our findings provide evidence that operation of these systems has been significantly impaired during the pandemic. More detailed exploration and analysis of the impact on the human rights of care home residents is warranted. A second concern pertains to the reported confusion about which legal framework applies to restrictive measures in locked‐down care homes. One of our most troubling findings is that the very professionals tasked with protecting the rights of exceptionally vulnerable residents found themselves unclear about what principles to apply in determining whether a particular restrictive measure complies with legal and human rights standards. Greater clarity is needed on how rights can and should be balanced against each other – both in times of crisis and in care homes’ settings more generally.

Finally, participants in our study reported longer‐standing issues surrounding the effective implementation of the MCA. Our findings reaffirm previously reported issues regarding the complexity and bureaucratic burden of MCA safeguarding processes (Carpenter et al., 2014) and inconsistent and variable knowledge of the MCA (Barry et al., 2020; Bartlett, 2014; House of Lords, 2014; Jayes et al., 2021; Lennard, 2015; Manthorpe & Samsi, 2016). The upcoming transition from the DoLS system to the new Liberty Protection Safeguards (LPS) presents a unique opportunity to ensure that the relevant provisions of law are well understood by the professionals who will play a leading role in implementing them.

## 5 LIMITATIONS

This study is limited by the size and non‐representative character of the sample, and by the self‐selecting participants who are likely to have been motivated to participate by their own experiences and perspectives on the issues being explored. Moreover, although the diverse range of capacity professionals represented provides a depth of perspective, most of our participants are not involved in the day‐to‐day care of residents. We also recognise that the voices of care home residents and their carers were not included this study. We call for further research exploring the experiences of residents, their carers, as well as professional groups underrepresented in this study.

## 6 CONCLUSION

This study sought to better understand how professionals perceived the impact and management of restrictive measures applied to residents with impaired mental capacity and how MCA safeguards designed to protect such residents operated during the pandemic. Our survey provides an overview of the perspectives and experiences of capacity professionals during the pandemic, whereas the focus groups provided an opportunity for in‐depth exploration. These professionals play a critical role in protecting the rights of people with impaired mental capacity, who have been among the most vulnerable to both the direct effects of COVID‐19 and the collateral consequences of its management. Although it is important to acknowledge the pressures faced by care homes during this period, the experiences reported in this article suggest that the impact of COVID‐19 for care home residents with impaired mental capacity has been varied but significant. We invite further exploration and analysis of (1) the variability and inconsistency of restrictions applied at care homes, (2) the strain placed on the MCA’s DoLS and advocacy systems, (3) the uncertainty about the remit of the MCA during a public health crisis and (4) the human rights implications of points (2) and (3). We also encourage (5) the development of appropriate training packages to address deficits in knowledge about the MCA.

## CONFLICT OF INTEREST

The authors declare that they have no conflict of interest.

## AUTHOR CONTRIBUTIONS

MK worked on the scoping review informing the survey, analysed webinar registration data, was the lead designer of the survey, developed the focus group protocol, facilitated the focus groups, anonymised focus group transcripts, filtered the data for this manuscript, was involved in all stages of data analysis for this manuscript and took the lead in writing the manuscript. AW contributed to the refinement of the survey questions and focus group protocol, attended focus groups, was involved in all stages of data analysis for this manuscript and made substantial contributions to the manuscript. VB contributed to the refinement of the survey questions and focus group protocol, attended the focus groups, was involved in the categorisation stage of data analysis for this article and provided feedback on the manuscript. EF worked on the scoping review informing the survey, identified key topics, contributed to the design of the survey and provided feedback on the manuscript. SM contributed to the refinement of the survey questions and focus group protocol, attended the focus groups, was involved in the categorisation stage of data analysis for this article and provided feedback on the manuscript. WM proposed and managed the study, contributed to the refinement of the survey questions and focus group protocol, attended the focus groups, was involved in the categorisation stage of data analysis for this article and made substantial contributions to the preparation of the manuscript.

## Supporting information

Supplementary Material

Supplementary Material

## Data Availability

Raw data are available via the UK Data Depository and will be held until November 2031.

## ENDNOTES

^1^
The MCA Code of Practice contains information about these roles (Department for Constitutional Affairs, 2007).

^2^
Roles included were: Advocates, BIAs, anyone working in a specialised dementia or learning disability facility or with a background in dementia or learning disability care, and anyone with ‘MCA’ or ‘Mental Capacity Act’,’ DoLS’, ‘capacity’, ‘LD’ or ‘Learning Disability’, ‘dementia’, and/or ‘CoP’ or ‘Court of Protection’ in their job title.

^3^
Outcomes of the broader project are available on the Essex Autonomy Project website: http://autonomy.essex.ac.uk/covid‐19/.

^4^
See Cairns et al. (2011) for documentation of an earlier version of this uncertainty.

## References

[hsc13747-bib-0001] Alzheimer’s Society . (2020). Thousands of people with dementia dying or deteriorating—Not just from coronavirus as isolation takes toll. Alzheimer’s Society. Available from: https://www.alzheimers.org.uk/news/2020‐06‐05/thousands‐people‐dementia‐dying‐or‐deteriorating‐not‐just‐coronavirus‐isolation

[hsc13747-bib-0002] Amnesty International . (2020). As if expendable: The UK government’s failure to protect older people in care homes during the COVID‐19 pandemic. Amnesty International. Available from: https://www.amnesty.org.uk/files/2020‐10/Care%20Homes%20Report.pdf

[hsc13747-bib-0003] Anka, A. , Thacker, H. , & Penhale, B. (2020). Safeguarding adults practice and remote working in the COVID‐19 era: Challenges and opportunities. The Journal of Adult Protection, 22(1), 415–427. 10.1108/jap-08-2020-0040

[hsc13747-bib-0041] Bartlett, P. (2014). Reforming the Deprivation of Liberty Safeguards (DOLS): What Is It Exactly that We Want? European Journal of Current Legal Issues, 20(3). http://webjcli.org/index.php/webjcli/article/view/355/465

[hsc13747-bib-0004] Barry, C. , Spathis, A. , Treaddell, S. , Carding, S. , & Barclay, S. (2020). Palliative care clinicians' knowledge of the law regarding the use of the Deprivation of Liberty Safeguards (DoLS). BMJ Supportive & Palliative Care, 10(2), e14. 10.1136/bmjspcare-2016-001186 28438759

[hsc13747-bib-0005] Bengtsson, M. (2016). How to plan and perform a qualitative study using content analysis. NursingPlus Open, 2, 8–14. 10.1016/j.npls.2016.01.001

[hsc13747-bib-0006] Brown, E. E. , Kumar, S. , Rajji, T. K. , Pollock, B. G. , & Mulsant, B. H. (2020). Anticipating and mitigating the impact of the COVID‐19 pandemic on Alzheimer's disease and related dementias. The American Journal of Geriatric Psychiatry, 28(7), 712–721. 10.1016/j.jagp.2020.04.010 32331845 PMC7165101

[hsc13747-bib-0007] Cairns, R. , Brown, P. , Grant‐Peterkin, H. , Khondoker, M. R. , Owen, G. S. , Richardson, G. , Szmukler, G. , & Hotopf, M. (2011). Judgements about deprivation of liberty made by various professionals: Comparison study. The Psychiatrist, 35(9), 344–349. 10.1192/pb.bp.110.033241

[hsc13747-bib-0008] Carpenter, J. , Langan, J. , Patsios, D. , & Jepson, M. (2014). Deprivation of liberty safeguards: What determines the judgements of Best Interests Assessors? A factorial survey. Journal of Social Work, 14(6), 576–593. 10.1177/1468017313504180

[hsc13747-bib-0009] Chu, C. H. , Donato‐Woodger, S. , & Dainton, C. J. (2020). Competing crises: COVID‐19 countermeasures and social isolation among older adults in long term care. Journal of Advanced Nursing, 76(10), 2456–2459. 10.1111/jan.14467 32643787 PMC7361866

[hsc13747-bib-0010] Courtenay, K. , & Perera, B. (2020). COVID‐19 and people with intellectual disability: Impacts of a pandemic. Irish Journal of Psychological Medicine, 37(3), 231–236. 10.1017/ipm.2020.45 32404232 PMC7287305

[hsc13747-bib-0011] Department for Constitutional Affairs . (2007). Mental Capacity Act 2005. Code of Practice, London, The Stationary Office. Available from: https://assets.publishing.service.gov.uk/government/uploads/system/uploads/attachment_data/file/921428/Mental‐capacity‐act‐code‐of‐practice.pdf

[hsc13747-bib-0012] Department of Health and Social Care . (2021). Guidance: Visiting arrangements in care homes. Available from: https://www.gov.uk/government/publications/visiting‐care‐homes‐during‐coronavirus

[hsc13747-bib-0013] Dunn, P. , Allen, L. , Alarilla, A. , Grimm, F. , Humphries, R. , & Alderwick, H. (2021). Adult social care and COVID‐19 after the first wave: Assessing the policy response in England. Our analysis of the national government policy response for social care between June 2020 and March 2021. Health Foundation. https://www.health.org.uk/publications/reports/adult‐social‐care‐and‐COVID‐19‐after‐the‐first‐wave

[hsc13747-bib-0015] Gordon, A. L. , Franklin, M. , Bradshaw, L. , Logan, P. , Elliott, R. , & Gladman, J. R. (2014). Health status of UK care home residents: A cohort study. Age and Ageing, 43(1), 97–103. 10.1093/ageing/aft077 23864424 PMC3861334

[hsc13747-bib-0017] House of Lords, Select Committee on the Mental Capacity Act 2005 . (2014). Mental Capacity Act 2005: Post‐legislative scrutiny. (HL 2013‐14 139). Available from: https://publications.parliament.uk/pa/ld201314/ldselect/ldmentalcap/139/13902.htm

[hsc13747-bib-0018] Hugelius, K. , Harada, N. , & Marutani, M. (2021). Consequences of Visiting restrictions during the COVID‐19 pandemic: An integrative review. International Journal of Nursing Studies, 121(104000), 104000. 10.1016/j.ijnurstu.2021.104000 34242976 PMC8196532

[hsc13747-bib-0019] Ivankova, N. V. , Creswell, J. W. , & Stick, S. L. (2006). Using mixed‐methods sequential explanatory design: From theory to practice. Field Methods, 18(1), 3–20. 10.1177/1525822X05282260

[hsc13747-bib-0020] Jayes, M. , Austin, L. , & Brown, L. J. E. (2021). Supported decision‐making and mental capacity assessment in care homes: A qualitative study. Health & Social Care in the Community, 1–9. 10.1111/hsc.13512 34250675

[hsc13747-bib-0042] Lennard, C. (2015). Deprivation of Liberty Safeguards (DoLS) – where do we go from here? The Journal of Adult Protection, 17(1), 41–50. 10.1108/jap-05-2014-0017

[hsc13747-bib-0021] Liu, K. Y. , Howard, R. , Banerjee, S. , Comas‐Herrera, A. , Goddard, J. , Knapp, M. , Livingston, G. , Manthorpe, J. , O'Brien, J. T. , Paterson, R. W. , Robinson, L. , Rossor, M. , Rowe, J. B. , Sharp, D. J. , Sommerlad, A. , Suárez‐González, A. , & Burns, A. (2021). Dementia wellbeing and COVID‐19: Review and expert consensus on current research and knowledge gaps. International Journal of Geriatric Psychiatry, 36(11), 1597–1639. 10.1002/gps.5567 34043836 PMC8237017

[hsc13747-bib-0022] Manthorpe, J. , & Samsi, K. (2016). Care homes and the Mental Capacity Act 2005: Changes in understanding and practice over time. Dementia, 15(4), 858–871. 10.1177/1471301214542623 25015949

[hsc13747-bib-0023] Marshall, F. , Gordon, A. , Gladman, J. R. , & Bishop, S. (2021). Care homes, their communities, and resilience in the face of the COVID‐19 pandemic: Interim findings from a qualitative study. BMC Geriatrics, 21(102), 1–10. 10.1186/s12877-021-02053-9 33546612 PMC7863040

[hsc13747-bib-0024] Ministry of Justice . (2008). Deprivation of liberty safeguards: Code of Practice to Supplement the Main Mental Capacity Act 2005. Code of Practice, London, The Stationery Office.

[hsc13747-bib-0025] Morgan, D. L. (2014). Pragmatism as a paradigm for social research. Qualitative Inquiry, 20(8), 1045–1053. 10.1177/1077800413513733

[hsc13747-bib-0026] O'Caoimh, R. , O'Donovan, M. R. , Monahan, M. P. , Dalton O'Connor, C. , Buckley, C. , Kilty, C. , Fitzgerald, S. , Hartigan, I. , & Cornally, N. (2020). Psychosocial impact of COVID‐19 nursing home restrictions on visitors of residents with cognitive impairment: A cross‐sectional study as part of the Engaging Remotely in Care (ERiC) project. Frontiers in Psychiatry, 11, 1115. 10.3389/fpsyt.2020.585373 PMC7649131 33192731

[hsc13747-bib-0027] Office of Public Sector Information (OPSI) . (2005). Mental Capacity Act 2005. OPSI.

[hsc13747-bib-0028] Parsons, J. A. , & Johal, H. K. (2020). Best interests versus resource allocation: Could COVID‐19 cloud decision‐making for the cognitively impaired? Journal of Medical Ethics, 46, 447–450. 10.1136/medethics-2020-106323 32376717 PMC7239662

[hsc13747-bib-0029] Pérez‐Rodríguez, P. , Díaz de Bustamante, M. , Aparicio Mollá, S. , Arenas, M. C. , Jiménez‐Armero, S. , Lacosta Esclapez, P. , González‐Espinoza, L. , & Bermejo Boixareu, C. (2021). Functional, cognitive, and nutritional decline in 435 elderly nursing home residents after the first wave of the COVID‐19 Pandemic. European Geriatric Medicine, 12(6), 1137–1145. 10.1007/s41999-021-00524-1 34165775 PMC8222945

[hsc13747-bib-0030] Public Health England . (2016). Learning disabilities observatory. People with learning disabilities in England 2015: Main report. Available from: https://assets.publishing.service.gov.uk/government/uploads/system/uploads/attachment_data/file/613182/PWLDIE_2015_main_report_NB090517.pdf

[hsc13747-bib-0032] Ruck Keene, A. (2020). Capacity in the time of Coronavirus. International Journal of Law and Psychiatry, 70, 101560. 10.1016/j.ijlp.2020.101560 32482298 PMC7151525

[hsc13747-bib-0033] Sizoo, E. M. , Monnier, A. A. , Bloemen, M. , Hertogh, C. M. , & Smalbrugge, M. (2020). Dilemmas with restrictive visiting policies in Dutch nursing homes during the COVID‐19 pandemic: A qualitative analysis of an open‐ended questionnaire with elderly care physicians. Journal of the American Medical Directors Association, 21(12), 1774–1781. 10.1016/j.jamda.2020.10.024 33197412 PMC7584414

[hsc13747-bib-0034] Tapper, J. (2020). Care home residents face postcode lottery over face‐to‐face visits. *The Guardian*. Available from: https://www.theguardian.com/society/2020/nov/07/care‐homes‐they‐dont‐put‐loneliness‐on‐death‐certificates‐but‐it‐is‐a‐killer

[hsc13747-bib-0035] Theis, N. , Campbell, N. , De Leeuw, J. , Owen, M. , & Schenke, K. C. (2021). The effects of COVID‐19 restrictions on physical activity and mental health of children and young adults with physical and/or intellectual disabilities. Disability and Health Journal, 14(3), 101064. 10.1016/j.dhjo.2021.101064 33549499 PMC7825978

[hsc13747-bib-0036] Vaismoradi, M. , Turunen, H. , & Bondas, T. (2013). Content analysis and thematic analysis: Implications for conducting a qualitative descriptive study. Nursing & Health Sciences, 15(3), 398–405. 10.1111/nhs.12048 23480423

[hsc13747-bib-0037] Velayudhan, L. , Aarsland, D. , & Ballard, C. (2020). Mental health of people living with dementia in care homes during COVID‐19 pandemic. International Psychogeriatrics, 32(10), 1253–1254. 10.1017/S1041610220001088 32487278 PMC7302947

[hsc13747-bib-0038] Vicary, S. , Stone, K. , McCusker, P. , Davidson, G. , & Spencer‐Lane, T. (2020). ‘It's about how much we can do, and not how little we can get away with’: Coronavirus‐related legislative changes for social care in the United Kingdom. International Journal of Law and Psychiatry, 72, 101601. 10.1016/j.ijlp.2020.101601 32889420 PMC7306708

[hsc13747-bib-0039] Wammes, J. D. , Kolk, D. , van den Besselaar, J. H. , MacNeil‐Vroomen, J. L. , Buurman‐van Es, B. M. , & van Rijn, M. (2020). Evaluating perspectives of relatives of nursing home residents on the nursing home visiting restrictions during the COVID‐19 crisis: A Dutch cross‐sectional survey study. Journal of the American Medical Directors Association, 21(12), 1746–1750. 10.1016/j.jamda.2020.09.031 33148480 PMC7524682

[hsc13747-bib-0040] Wilson, K. (2020). The COVID‐19 pandemic and the human rights of persons with mental and cognitive impairments subject to coercive powers in Australia. International Journal of Law and Psychiatry, 73, 101605. 10.1016/j.ijlp.2020.101605 33157404 PMC7318936

