# Supplementary material for: COVID‐19 and the Mental Capacity Act in care homes: Perspectives from capacity professionals
Source: Health Soc Care Community. 2022 Feb 9:10.1111/hsc.13747. Online ahead of print. doi: 10.1111/hsc.13747 (PMC9111706; doi:10.1111/hsc.13747)
Supplement: Supplementary file 1 — Supplementary Material [file HSC-9999-0-s002.docx]

Appendix- A

Human Rights in Care Homes: Survey instrument

Survey flow

Block: Default Question Block (2 questions)

Standard: General information (10 questions)

Branch: New branch

If

If which role(s) do you have? Tick all that apply. Paramedic is not selected.

Standard: Restrictive measures (16 questions)

Standard: Use of guidance (6 questions)

Standard: Access to services (7 questions)

Branch: New branch

If

If which role(s) do you have? Tick all that apply. Paramedic is not selected.

Standard: Access to services continued (3 questions)

Standard: Use of DNACPR orders (9 questions)

Standard: Block 7 (1 question)

| Page Break |  |
| --- | --- |

Start of Block: Default Question Block

Q1 Human Rights in Care Homes: A Survey-Based Study    We are inviting you to participate in this survey so we can learn about the experiences of professionals working in or with care homes during the Covid-19 pandemic.    This survey is part of a larger research project, ‘Human Rights in Care Homes’, focusing on the impact of the Covid-19 pandemic on respect for human rights in care homes. Our goal is to help care professionals and policy makers protect human rights in care homes going forward. By completing this survey, you will help us understand the situation on the ground and what support may be needed.    Though the survey is primarily targeted at people working in England or Wales, we welcome responses from professionals working elsewhere. We would like to reassure you that the survey is completely anonymous, meaning we will not collect any information that can identify you. You can also withdraw at any time by simply closing your browser. Please make sure you have read the [participant information sheet](https://autonomy.essex.ac.uk/wp-content/uploads/2021/03/Survey-Participant-Information-Sheet.pdf) before proceeding to the survey. 

**Note: This survey will close at Midnight on** **Sunday, April 4, 2021.**

- I understand that, by completing this survey, I am consenting to take part in this study. (4)

| Page Break |  |
| --- | --- |

Q2 Thank you for agreeing to take part in this survey. Depending on your professional role, you will be asked questions about some or all of the following topics:

Restrictions on movement and visits

The usefulness of guidance

Access to services

The use of DNACPR (Do Not Attempt Cardio-Pulmonary Resuscitation) orders

**We understand that some of the questions may be quite sensitive, so we would like to thank you in advance for your willingness to help.**

End of Block: Default Question Block

Start of Block: General information

Q3
**General information**
 
First of all, we would like to ask you some general questions about where you work and what you do.

Q4 In which region do you work?

- England (1)
- Wales (2)
- Other (please specify) (3) ________________________________________________
- Prefer not to say (4)

| Page Break |  |
| --- | --- |

Display This Question:

If Q4 = England

Q5 In which area of England do you work?

- South East (1)
- London (2)
- North West (3)
- East of England (4)
- West Midlands (5)
- South West (6)
- Yorkshire and the Humber (7)
- East Midlands (8)
- North East (9)
- Prefer not to say (10)

| Page Break |  |
| --- | --- |

Q6 Which role(s) do you have? Tick all that apply.

- Facility Management Team member (e.g. Care Home Manager or Director) (1)
- Social worker (2)
- Care Home Nurse/Carer (3)
- Primary Care Clinical Lead for a care home (e.g. named GP) (4)
- Advocate (e.g. IMCA or RPR) (5)
- Best Interests Assessor (BIA) (6)
- Paramedic (9)
- Other (please specify) (7) ________________________________________________
- ⊗Prefer not to say (8)

Display This Question:

If If Which role(s) do you have? Tick all that apply. q://QID8/SelectedChoicesCount Is Greater Than 1

Carry Forward Selected Choices from ‘Q6’

| 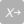 |
| --- |

Q7 What do you consider to be your **primary** role?

- Facility Management Team Member (e.g. Care Home Manager or Director) (1)
- Social worker (2)
- Care Home Nurse/Carer (3)
- Primary Care Clinical Lead for a care home (e.g. named GP) (4)
- Advocate (e.g. IMCA or RPR) (5)
- Best Interests Assessor (BIA) (6)
- Paramedic (7)
- Other (please specify) (8) ________________________________________________
- ⊗Prefer not to say (9)

| Page Break |  |
| --- | --- |

Display This Question:

If Q6 != Paramedic

Q8 Since the onset of the pandemic in March 2020, have you...

- ...worked mainly within care homes (physically present) (1)
- ...worked mainly with care homes from the outside (remotely, e.g. conducting remote DoLS authorisations) (2)
- My professional role has not involved working within or with care homes during the pandemic (3)
- Prefer not to say (4)

| Page Break |  |
| --- | --- |

Display This Question:

If Q8 = My professional role has not involved working within or with care homes during the pandemic

Q9 Given the focus of our study, we are only collecting responses from people who have worked in or with care homes during the pandemic. Many thanks for your interest.

Skip To: End of Survey If Q9 Is Displayed

| Page Break |  |
| --- | --- |

Q10 Since the onset of the pandemic in March 2020, have you...

- ...worked mainly in or with one care home (1)
- ... worked in or with several care homes (2)
- Not sure (3)
- Prefer not to say (4)

| Page Break |  |
| --- | --- |

Display This Question:

If Q10 = ...worked mainly in or with one care home

Q11 How many residents live at this care home?

- 1-10 residents (1)
- 11-50 residents (5)
- 51-100 residents (6)
- 100+ residents (7)
- Not sure (2)
- Prefer not to say (4)

| Page Break |  |
| --- | --- |

Display This Question:

If Q6 != Paramedic

Q12 At the care home(s) you worked in or with during the pandemic, were there any residents with **impaired decision-making capacity?**

- Yes (1)
- No (2)
- Not sure (3)
- Prefer not to say (4)

End of Block: General information

Start of Block: Restrictive measures

Q13
Restrictions on visits and movement 
  We would like to learn more about the restrictive measures that might have been implemented at care homes during the pandemic.

Q14 At the care home(s) you worked in or with during the pandemic, were any restrictions to **visits** implemented (e.g. only window or outdoors visits permitted)?

- Yes (1)
- No (2)
- Not sure (3)
- Prefer not to say (4)

Skip To: Q17 If Q14 = No

Skip To: Q17 If Q14 = Not sure

Skip To: Q17 If Q14 = Prefer not to say

| Page Break |  |
| --- | --- |

Q15 Which measures were implemented to restrict visits? Tick any measures that you saw implemented, at the care home(s) you worked in or with, at any point in time during the pandemic.

- Limited number of visitors permitted per resident (1)
- Only window visits permitted (2)
- Only outside visits permitted (3)
- Socially distanced visits allowed with supervision or barriers (e.g. plexiglass) (4)
- Visits with PPE permitted (5)
- No visits allowed for some residents (6)
- No visits allowed whatsoever (7)
- Other (please specify) (8) ________________________________________________
- ⊗Not sure (9)
- ⊗Prefer not to say (10)

Skip To: Q17 If Q15 = Not sure

Skip To: Q17 If Q15 = Prefer not to say

| Page Break |  |
| --- | --- |

Carry Forward Selected Choices from ‘Q15’

| 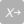 |
| --- |

Q16 Which of these measures to restrict visits to residents are **still in place** at this point in time (March 2021)? Tick all that apply.

- ⊗None of these measures are still in place (1)
- Limited number of visitors permitted per resident (2)
- Only window visits permitted (3)
- Only outside visits permitted (4)
- Socially distanced visits allowed with supervision or barriers (e.g. plexiglass) (5)
- Visits with PPE permitted (6)
- No visits allowed for some residents (7)
- No visits allowed whatsoever (8)
- Other (please specify) (9) ________________________________________________
- ⊗Not sure (10)
- ⊗Prefer not to say (11)

| Page Break |  |
| --- | --- |

Q17 At the care home(s) you worked in or with during the pandemic, were any restrictions on**movement**implemented (for example, residents not being allowed to exit the facility)?

- Yes (1)
- No (2)
- Not sure (3)
- Prefer not to say (4)

Skip To: End of Block If Q17 = No

Skip To: End of Block If Q17 = Not sure

Skip To: End of Block If Q17 = Prefer not to say

Q18 Which measures were implemented to restrict the **movement** of residents? Tick any measures that you saw implemented, at the care home(s) you worked in or with, **at any point in time** during the pandemic.

- Restriction to room of some residents (2)
- Restriction to room of all residents (1)
- Restriction to a specific area within the facility of all residents (3)
- Restriction to a specific area within the facility of some residents (4)
- Residents not allowed to exit facility (5)
- Residents not allowed to leave grounds of facility (6)
- Other (please specify) (7) ________________________________________________
- ⊗Not sure (8)
- ⊗Prefer not to say (9)

Skip To: Q26 If Q18 = Not sure

Skip To: Q26 If Q18 = Prefer not to say

Carry Forward Selected Choices from ‘Q18’

| 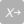 |
| --- |

Q19 Which of these measures to restrict the movement of residents are **still in place** **at this point in time** (March 2021)?

- ⊗None of these measures are still in place (1)
- Restriction to room of some residents (2)
- Restriction to room of all residents (3)
- Restriction to a specific area within the facility of all residents (4)
- Restriction to a specific area within the facility of some residents (5)
- Residents not allowed to exit facility (6)
- Residents not allowed to leave grounds of facility (7)
- Other (please specify) (8) ________________________________________________
- ⊗Not sure (9)
- ⊗Prefer not to say (10)

| Page Break |  |
| --- | --- |

Display This Question:

If Q18 = Restriction to room of all residents

Or Q18 = Restriction to room of some residents

Q20 What was, approximately, **the longest period of time** that you saw a resident being restricted to their room?

- Less than a week (1)
- 1-2 weeks (2)
- 2-4 weeks (3)
- Over 4 weeks (4)
- Not sure (5)
- Prefer not to say (6)

| Page Break |  |
| --- | --- |

Display This Question:

If Q18 = Restriction to room of some residents

And Q18 != Restriction to room of all residents

Q21 In your experience, on what basis were residents confined to their room? Tick all that apply.

- Resident was confirmed or suspected COVID-19 case (1)
- Resident had been in contact with confirmed or suspected COVID-19 case (3)
- Resident was newly admitted to the facility (7)
- Resident struggled to adhere to social distancing (e.g. due to dementia) (8)
- Resident was clinically vulnerable (9)
- Other (please specify) (10) ________________________________________________
- Not sure (4)
- Prefer not to say (5)

| Page Break |  |
| --- | --- |

Q22 When residents were restricted to their room, what was generally the most important consideration?

- Protecting other residents and staff (1)
- Best interests of the resident (2)
- Other (please specify) (3) ________________________________________________
- I don't know (4)
- Prefer not to say (5)

| Page Break |  |
| --- | --- |

Display This Question:

If Q12 = Yes

And If

Q18 = Restriction to room of some residents

Or Q18 = Restriction to room of all residents

Q23 At the care home(s) you worked in or with during the pandemic, were any residents with impaired decision-making capacity confined to their room?

- Yes (1)
- No (2)
- Not sure (3)
- Prefer not to say (4)

| Page Break |  |
| --- | --- |

Display This Question:

If Q23 = Yes

Q24 When residents with impaired decision-making capacity were confined to their room, how often was a new **DoLS (Deprivation of Liberty Safeguards)** authorisation provided?

- Always or most of the time (1)
- Often (more than half of the time) (2)
- Sometimes (less than half of the time) (3)
- Rarely or never (4)
- Not sure (5)
- Prefer not to say (6)

| Page Break |  |
| --- | --- |

Display This Question:

If Q24 = Often (more than half of the time)

Or Q24 = Sometimes (less than half of the time)

Or Q24 = Rarely or never

Q25 When a new DoLS authorisation was **not** provided, this was **usually** because...

- ...there was already a standard DoLS authorisation in place for these residents (1)
- ...residents were confined to their room in response to public health directives rather than under the Mental Capacity Act (2)
- ...it was not a priority under the circumstances (3)
- ...it was not feasible (e.g. time or staff shortages) (4)
- ...of another reason (please explain) (5) ________________________________________________
- Not sure (6)
- Prefer not to say (7)

| Page Break |  |
| --- | --- |

Q26 Were you involved in any decisions to restrict visits **and/or** movement at care homes during the pandemic?

- Yes (1)
- No (2)
- Not sure (3)
- Prefer not to say (4)

| Page Break |  |
| --- | --- |

Q27 In your experience, how have residents and/or family members of residents generally responded to these restrictions (on visits **and/or** movement)?

- They were generally supportive (1)
- Mixed – some were supportive, others objected (2)
- They generally objected (3)
- Not sure (4)
- Prefer not to say (5)

| Page Break |  |
| --- | --- |

Q28 In your experience, what has been the general impact of these restrictions (on visits **and/or movement**) on residents?

- I have noticed a positive impact, for example, residents felt more safe (3)
- I have noticed a slight negative impact, for example, mild symptoms of increased anxiety, depression or cognitive impairments among residents (2)
- I have noticed a significant negative impact, for example, severe symptoms of increased anxiety, depression or cognitive impairments among residents (1)
- I have not noticed any significant impact (4)
- Not sure (5)
- Prefer not to say (6)

End of Block: Restrictive measures

Start of Block: Use of guidance

Q29
**Usefulness of guidance**
 
We would also like to learn more about how useful **guidance** has been for practice in and with care homes during the pandemic.

Q30 At the **current stage of the pandemic** (January 2021–now), do you think there is....

- ...too much guidance for practice in care homes (1)
- ...about enough guidance for practice in care homes (2)
- ...not enough guidance for practice in care homes (3)
- ...not enough adequate guidance for practice in care homes (4)
- Not sure (5)
- Prefer not to say (6)

| Page Break |  |
| --- | --- |

Display This Question:

If Q30 = ...not enough guidance for practice in care homes

Q31 What would you like to see more guidance on? 


If you are not sure or would prefer not to answer, you can click ‘Next’.

________________________________________________________________

| Page Break |  |
| --- | --- |

Display This Question:

If Q30 = ...not enough adequate guidance for practice in care homes

Q32 In your opinion, how could guidance be made more adequate? 


If you are not sure or would prefer not to answer, you can click ‘Next’.

________________________________________________________________

| Page Break |  |
| --- | --- |

Q33 In your experience, at the current stage of the pandemic (January–March 2021), care home staff generally...

- ...follow guidance as strictly as possible (1)
- ....follow guidance loosely (2)
- ...feel unsure about which guidance to follow (3)
- ...do not follow guidance (please explain why) (4) ________________________________________________
- Not sure (5)
- Prefer not to say (6)

| Page Break |  |
| --- | --- |

Q34 In your experience, which guidance has been relied upon by care homes since the onset of the pandemic? Tick all that apply.

- Local guidance (e.g. issued by Local Authority, care home network, Trust) (1)
- National guidance (issued by National Government/Department of Health and Social Care) (2)
- National Institute for Health and Care Excellence (NICE) guidelines (3)
- Social Care Institute for Excellence (SCIE) guidelines (4)
- Royal colleges (e.g. Royal College of Nursing guidance) (5)
- Care home representative groups guidance (e.g. Care England) (10)
- Other societies or organisations (e.g. British Geriatrics Society, Skills For Care) (6)
- Other guidance (Please specify) (7) ________________________________________________
- ⊗Not sure (8)
- ⊗Prefer not to say (9)

End of Block: Use of guidance

Start of Block: Access to services

Q35
**Access to services**
  We would like to understand how the pandemic has affected **access to services** for care home residents.

Q36 At the care home(s) you worked in or with during the pandemic, have residents been able to access **in-person GP care** when this was needed?

- Yes, unless there was a COVID-19 outbreak at the care home (1)
- Yes, always (2)
- Often (more than half of the time) (3)
- Sometimes (less than half of the time) (4)
- Rarely or never (5)
- Not sure (6)
- Prefer not to say (7)

| Page Break |  |
| --- | --- |

Q37 At the care home(s) you worked in or with during the pandemic, have residents been able to access other types of in-person health care (e.g. community nurses) when this was needed?

- Yes, unless there was a COVID-19 outbreak at the care home (1)
- Yes, always (2)
- Often (more than half of the time) (3)
- Sometimes (less than half of the time) (4)
- Rarely or never (5)
- Not sure (6)
- Prefer not to say (7)

| Page Break |  |
| --- | --- |

Q38 Through your work in or with care homes during the pandemic, were you involved in any decisions about whether or not to transfer residents to hospital?

- Yes (1)
- No (2)
- Not sure (3)
- Prefer not to say (4)

| Page Break |  |
| --- | --- |

Q39 There have been reports that some care home residents were not transferred to hospital because of the pandemic (for example because there were no ambulances available). 


At the care home(s) you worked in or with during the pandemic, were any residents **not** transferred to hospital **who would have been transferred to hospital under normal circumstances?**

- Yes (1)
- No (2)
- Not sure (3)
- Prefer not to say (4)

Skip To: End of Block If Q39 = No

Skip To: End of Block If Q39 = Not sure

Skip To: End of Block If Q39 = Prefer not to say

| Page Break |  |
| --- | --- |

Q40
We would like to learn on what basis those residents were not transferred to hospital, as this can be due to many reasons.
 
In your experience, when residents were not transferred to hospital who would have been transferred under normal circumstances, on what basis did this happen? Tick all that apply.

- Risk that resident would contract COVID-19 at hospital (2)
- Hospital was not accepting patients (6)
- No ambulances were available (7)
- It was decided not to transfer any residents with a DNACPR order to hospital (3)
- It was decided not to transfer any residents with certain characteristics (e.g. age, frailty) to hospital (4)
- It was decided not to transfer any residents to hospital whatsoever (5)
- GP did not refer resident (12)
- Staff or time shortage (8)
- Other (please specify) (9) ________________________________________________
- ⊗Not sure (10)
- ⊗Prefer not to say (11)

Display This Question:

If Q40 = It was decided not to transfer any residents with certain characteristics (e.g. age, frailty) to hospital

Q41 When it was decided not to transfer any residents with certain characteristics to hospital (who would have been transferred under normal circumstances), on the basis of which characteristics did this happen? Tick all that apply.

- Age (1)
- Frailty (2)
- Dementia/cognitive impairment (3)
- Other pre-existing conditions (4)
- Other (please specify) (5)
- ⊗Not sure (6)
- ⊗Prefer not to say (7)

End of Block: Access to services

Start of Block: Access to services continued

Display This Question:

If Q12 = Yes

Q42 We also have a few questions about the role of **IMCAs** (Independent Mental Capacity Advocates) during the pandemic. In your experience, were care homes generally able to **access** an IMCA when one was needed during the pandemic?

- Yes – most of the time (also outside of business hours) (1)
- Yes – but only during business hours (2)
- No (3)
- Not sure (4)
- Prefer not to say (5)

| Page Break |  |
| --- | --- |

Display This Question:

If Q12 = Yes

Q43 In your experience, what was the **most** **common** mode of access for IMCAs at care homes during the pandemic?

- In person (1)
- Via telephone (2)
- Via video call (e.g. Zoom) (3)
- Other (please specify) (4) ________________________________________________
- Not sure (5)
- Prefer not to say (6)

| Page Break |  |
| --- | --- |

Display This Question:

If Q12 = Yes

Q44 In your experience, the involvement of IMCAs during the pandemic has generally been:

- An aid to effective best interests decision-making (Please explain why) (1) ________________________________________________
- Neither an aid nor a hindrance to best interests decision-making (2)
- A hindrance to best interests decision-making (Please explain why) (3) ________________________________________________
- Not sure (6)
- Prefer not to say (7)

End of Block: Access to services continued

Start of Block: Use of DNACPR orders

Q45
**Use of DNACPR orders**
 
Finally, we would like to learn more about how the pandemic has affected the use of **DNACPR** (Do Not Attempt Cardio-Pulmonary Resuscitation) orders in care homes.

Q46 One thing we would like to learn is whether the pandemic has affected practices of **adding** DNACPR orders to the files of care home residents. 


At the care home(s) you worked in or with during the pandemic, were new DNACPR orders added to the files of residents?

- Yes, new DNACPR orders were added during the pandemic (1)
- No, there were no DNACPR orders added that I know of (2)
- Not sure (3)
- Prefer not to say (4)

Skip To: Q51 If Q46 = Not sure

Skip To: Q51 If Q46 = No, there were no DNACPR orders added that I know of

Skip To: Q51 If Q46 = Prefer not to say

| Page Break |  |
| --- | --- |

Q47 Why were DNACPR orders added to the files of residents? Tick all that apply.

- For reasons unrelated to the pandemic (e.g. due to a general review or new guidance) (1)
- Because the pandemic highlighted the need for (re)considering DNACPR orders (2)
- Upon resident or family request in response to the pandemic (3)
- Other (please specify) (4) ________________________________________________
- ⊗Not sure (5)
- ⊗Prefer not to say (6)

| Page Break |  |
| --- | --- |

Q48 Were you involved in any decisions about adding DNACPR orders to the files of residents?

- Yes (1)
- No (2)
- Not sure (3)
- Prefer not to say (4)

| Page Break |  |
| --- | --- |

Q49 There have been reports that DNACPR orders were added to the files of care home residents without consultation with the resident or their family. 


Through your work in or with care homes during the pandemic, have you witnessed any DNACPR orders being added without consultation with the resident or their family?

- No, residents or their family were always consulted when a DNACPR order was added (1)
- Yes, sometimes a DNACPR order was added without consulting the resident or their family (2)
- Not sure (3)
- Prefer not to say (4)

| Page Break |  |
| --- | --- |

Display This Question:

If Q49 = Yes, sometimes a DNACPR order was added without consulting the resident or their family

Q50 In your experience, when DNACPR orders were added to the files of residents without consulting the resident or their family, on what basis did this happen? Tick all that apply.

- It was decided to add a DNACPR order to the file of all new residents upon admission to the care home (1)
- It was decided to add a DNACPR order to the file of a certain group of residents, e.g. above a certain age (2)
- It was decided to add a DNACPR order to the file of all residents across the care home (3)
- Other (please specify) (4) ________________________________________________
- ⊗Not sure (5)
- ⊗Prefer not to say (6)

| Page Break |  |
| --- | --- |

Display This Question:

If Q50 = It was decided to add a DNACPR order to the file of a certain group of residents, e.g. above a certain age

Q50 When DNACPR orders were added to the files of a certain group of residents, on the basis of which characteristics did this happen? Tick all that apply.

- Age (1)
- Frailty score (2)
- Dementia/cognitive impairment (3)
- Other pre-existing conditions (4)
- Other (please specify) (5) ________________________________________________
- ⊗Not sure (6)
- ⊗Prefer not to say (7)

| Page Break |  |
| --- | --- |

Q51 We would also like to learn whether the pandemic changed the way DNACPR orders were **used**. There have been reports that DNACPR orders sometimes influenced medical decisions beyond CPR. 

 At the care home(s) you worked in or with during the pandemic, have you witnessed DNACPR orders influence any medical decisions beyond CPR **at any point in time** since the onset of the pandemic?

- No, DNACPR orders only influenced decisions about CPR (1)
- Yes, sometimes DNACPR orders influenced other medical decisions (please specify which) (2) ________________________________________________
- Not sure (3)
- Prefer not to say (4)

| Page Break |  |
| --- | --- |

Display This Question:

If Q51 = Yes, sometimes DNACPR orders influenced other medical decisions (please specify which)

Q52 At the care home(s) you worked in or with during the pandemic, are you aware of DNACPR orders influencing medical decisions beyond CPR at this point in time (March 2021)?

- Yes (please specify which decisions) (1) ________________________________________________
- No (2)
- Not sure (3)
- Prefer not to say (4)

End of Block: Use of DNACPR orders

Start of Block: Block 7

Q53 You have nearly made it to the end of the survey! Is there anything else you want to tell us about your experiences working in or with care homes during the pandemic?


If you do not have anything else to tell us, you can click ‘Next’.

________________________________________________________________

________________________________________________________________

________________________________________________________________

________________________________________________________________

________________________________________________________________

End of Block: Block 7
