# Supplementary material for: COVID‐19 and the Mental Capacity Act in care homes: Perspectives from capacity professionals
Source: Health Soc Care Community. 2022 Feb 9:10.1111/hsc.13747. Online ahead of print. doi: 10.1111/hsc.13747 (PMC9111706; doi:10.1111/hsc.13747)
Supplement: Supplementary file 2 — Supplementary Material [file HSC-9999-0-s001.docx]

# Appendix B: Focus Groups Protocol

Researchers involved in conducting the focus groups: Dr Margot Kuylen, Dr Aaron Wyllie, Dr. Vivek Bhatt, Prof. Wayne Martin, Prof. Sabine Michalowski

## Introduction

- Welcome statement
- Introduction of researchers present
- Introduction of participants: participants are asked to introduce themselves by describing their job role (without naming their employer) and stating whether they worked within or rather with care homes during the pandemic.
- Give more information about the session:
  - remind participants that data will be transcribed and anonymised, and that session is confidential – remind participants not to discuss what is being said with anyone not present and that they can leave their camera off if they so wish;
  - remind participants that data will be stored on a secure drive with limited access;
  - notify participants that we cannot control what Zoom does with the data;
  - explain that participants have permission to leave the session at any time without giving a reason;
  - ask participants to
    - mute themselves unless they are speaking;
    - raise their hand if they want to say something and
    - remain respectful of others during the session.
  - share the agenda: which topics will be covered during the session;
  - ask participants to be as specific as possible when giving their answers, including examples and stories where possible;
  - emphasise that participants should feel free to ask if anything is unclear.

## Theme 1: Access to care

- Our survey indicates there were issues with access to GP care during the pandemic. Was this your experience?
- Can somebody give a concrete example of poor, or very good, access?
  - Follow-up options:
    - If there was poor access, what were the obstacles?
    - If there was good access, what made it work well?
    - Before the pandemic, was it easy to access GP care when this was needed?
    - What happened when in-person GP care could not be accessed, and what impact did this have on things like resident well-being, staff workloads?
- How was access to other types of healthcare?

## Theme 2: Restrictive measures

- Our survey showed that making decisions about restrictions on movement and visits involved balancing many conflicting interests – for example, balancing individual rights with the need to protect residents and staff. Can somebody describe how these interests were balanced when decisions about restrictions were made?
- Can somebody give an example of a situation where, in your experience, the right balance was (not) struck?
  - Follow-up options:
    - In this situation, were less restrictive alternatives considered?
    - (How) was the situation reconsidered?
- Our survey indicated also indicated that new DoLS authorisations were very rarely added during the pandemic and also that many people were unsure about whether they were added. Can you talk to us a little bit about how DoLS authorisations were handled?
- Follow-up options:
  - - Was it at all discussed whether new ones were needed?
    - On what basis were decisions made about whether or not to apply for a new authorisation?
    - How *should* the DoLS system work during a pandemic? Is it the right framework to handle restrictions in this type of crisis?

## Theme 3: DNACPR orders

- We would like to ask you some questions about DNACPR orders. First of all, we want to understand the process of adding them to a resident’s file, and whether the pandemic has affected this process. Could somebody talk us through the usual process of adding an order to a file?
  - Follow-up option: Who is normally involved in decisions about DNACPRs?
- In your experience, has the process of adding them changed over the course of the pandemic?
  - Follow-up option: Our survey shows that the pandemic highlighted the need to think about, or rethink, DNACPR orders. Why do you think this was the case?
- We would also like to understand how DNACPR orders are *used,* what they mean in practice. Our survey shows that, during the pandemic, DNACPR orders sometimes influenced medical decisions beyond CPR. Can anyone give us an example of a situation in which this happened?
  - Follow-up options if examples are given:
    - How do you know this happened? (Witness or hearsay)
    - Did this happen only during the pandemic or also before?
    - Was there a change in how these orders were used over the course of the pandemic?
- Does anyone have experience with the ReSPECT form?
  - Follow-up option: if so, how does it compare to the DNACPR form?

## Theme 4: IMCAs

- **Our survey data shows that people were somewhat divided on the effectiveness of IMCAs. When we removed answers by IMCAs, opinions were even more divided.** How would you make sense of this diversity of opinion?
  - Follow-up option: Is this due to the pandemic or a more general issue?
- Can somebody give an example of IMCA involvement (not) working well?
- How could IMCAs be more effective?
